# Development of a Web-Based System to Report Medication-Related Adverse Effects: Design and Usability Study

**DOI:** 10.2196/37605

**Published:** 2022-10-07

**Authors:** Renly Lim, Christopher Thornton, Jan Stanek, Lisa Kalisch Ellett, Myra Thiessen

**Affiliations:** 1 Quality Use of Medicines and Pharmacy Research Centre UniSA Clinical and Health Sciences University of South Australia Adelaide Australia; 2 UniSA Creative University of South Australia Adelaide Australia; 3 UniSA STEM University of South Australia Adelaide Australia; 4 Art, Design and Architecture Monash University Melbourne Australia

**Keywords:** adverse drug reaction, adverse drug event, digital health, eHealth, medication safety, mHealth, participatory design, patient reported outcomes, telehealth

## Abstract

**Background:**

Medicine use is the most common intervention in health care. The frequency with which medicines are used means medication-related problems are very common. One common type of medication-related problems is adverse drug events, which are unintended and harmful effects associated with use of medicines. Reporting of adverse drug events to regulatory authorities is important for evaluation of safety of medicines; however, these adverse effects are frequently unreported due to various factors, including lack of consumer-friendly reporting tools.

**Objective:**

The aim of this study was to develop a user-friendly digital tool for consumers to report medication-related adverse effects.

**Methods:**

The project consisted of 3 parts: (1) content development, including a systematic literature search; (2) iterative system development; and (3) usability testing. The project was guided by participatory design principles, which suggest involving key stakeholders throughout the design process. The first 2 versions were developed as a mobile app and were tested with end users in 2 workshops. The third version was developed as a web application and was tested with consumers who were taking regular medicines. Consumers were asked to complete a modified version of the mHealth app usability questionnaire (MAUQ), an 18-item questionnaire with each item scored using a 7-point Likert scale ranging from 0 (strongly disagree) to 7 (strongly agree). The MAUQ assessed 3 subscales including ease of use (5 items), interface and satisfaction (7 items), and usefulness (6 items). Continuous variables were reported as mean (SD) values, whereas categorical variables were presented as frequencies (percentages). Data analysis was conducted in Microsoft Excel.

**Results:**

The content for the system was based on a systematic literature search and short-listing of questions, followed by feedback from project team members and consumers. Feedback from consumers in the 2 workshops were incorporated to improve the functionality, visual design, and stability of the third (current) version. The third version of the system was tested with 26 consumers. A total of 79% (N=307/390) of all responses on the MAUQ were scored 6 or 7, indicating that users generally strongly agree with the usability of the system. When looking at the individual domains, the system had an average score of 6.3 (SD 0.9) for “ease of use,” 6.3 (SD 0.8) for “interface and satisfaction,” and 5.2 (SD 1.4) for “usefulness.”

**Conclusions:**

The web-based system for medicine adverse effects reporting is a user-friendly tool developed using an iterative participatory design approach. Future research includes further improving the system, particularly the usefulness of the system, as well as testing the scalability and performance of the system in practice.

## Introduction

Adverse drug events refer to the unintended and harmful effects associated with the use of medicines. Adverse drug reaction (ADR) is a subset of adverse drug events, where there is a causal relationship between the medicines and the adverse effects [1]. Adverse drug events add a significant burden to the health care system due to increased hospital admissions or emergency department visits; prolonged hospital stays; more complex patient management; complications, including disability and death; and potential prescribing cascade, where another medication is prescribed to ‘treat’ the adverse effects [2-6]. In Australia, we estimate that approximately 250,000 hospital admissions annually are medicine-related, costing AUD $1.4 billion annually [7-9], and that at least one million people have experienced an ADR in the past 6 months [7].

Although some medication-related adverse effects such as fatigue and dizziness may not be considered serious, these adverse effects can have a profound impact on patients’ quality of life [5,10]. Further, these seemingly mild adverse effects have the potential to lead to more serious adverse events, such as falls, fractures, and hospitalizations. Recognizing and preventing medication-related adverse effects is therefore important, so that patients do not suffer from avoidable harms, with more serious adverse events also being preventable, resulting in fewer complications and a reduction in health care costs.

At the population level, reporting of ADRs to regulatory authorities is crucial for effective safety monitoring of medicines. Reporting rates of ADRs are however very low; up to 95% of ADRs are not reported to regulatory authorities [11]. In Australia, reporting of serious ADRs and adverse events to regulatory authorities is mandatory for pharmaceutical companies but voluntary for health care professionals and patients. Most of ADR reports are made by pharmaceutical companies, about 20% are from health care professionals, and less than 5% are from patients [12].

Many interventions have been developed to improve ADR reporting rates but have shown mixed results [13]. Most interventions targeted only health care professionals [13]. Reports from patients are increasingly recognized as important sources of information about ADRs [14]. Previous studies have shown that patients are able to detect ADRs first [15,16], and that patient ADR reports alert regulatory authorities to new and previously unknown ADRs [17]. However, barriers such as lack of awareness of the importance of ADR reporting, difficulty using existing reporting systems due to complicated language and cumbersome interfaces, and negative reporting experiences mean that ADR reporting rates from patients remain very low [11,18,19].

The aim of our study was to develop a user-friendly digital tool (SideRep) to report medication-related adverse effects, primarily for consumer use. This paper describes the development, design, and usability of the SideRep system.

## Methods

**Figure 1:**
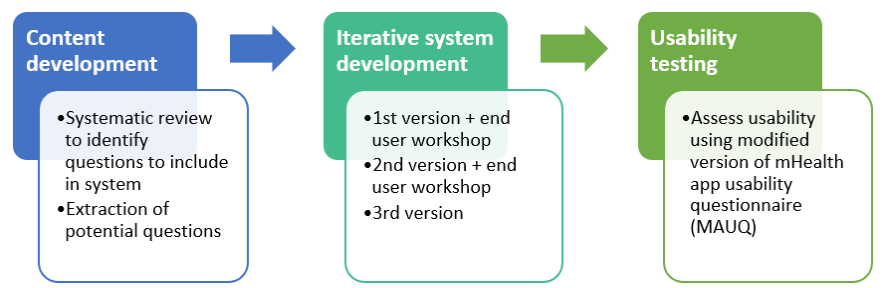
Processes involved during content development, system development, and usability testing.

The SideRep project consisted of 3 parts: (1) content development, (2) iterative system development, and (3) usability testing (Figure 1). The 3 phases were conducted in South Australia between January and August 2020. The project was guided by participatory design principles [20], which suggest involving key stakeholders throughout the design process. Our project team consisted of researchers with backgrounds in communication design, pharmacy, and software development.

### Content Development

The purpose of the project was to develop a simple and robust system for consumers to report any medication-related side effects that they felt. The first step of the project was therefore to determine relevant questions that should be included in the system. A systematic literature review was carried out to identify available consumer-reported medicine side effects questionnaires [21]. Variables considered relevant to our app content development were extracted, including the number and types of questions asked, the use of scoring system, the presence of open-ended questions, and the time taken to complete the questionnaire. One study investigator (RL, a clinical pharmacist) reviewed all short-listed questionnaires to extract possible questions for inclusion in our system, with the understanding that the questions may change following feedback from project team members and consumers during the second part of the study (ie, iterative system development).

### Iterative System Development

**Figure 2:**
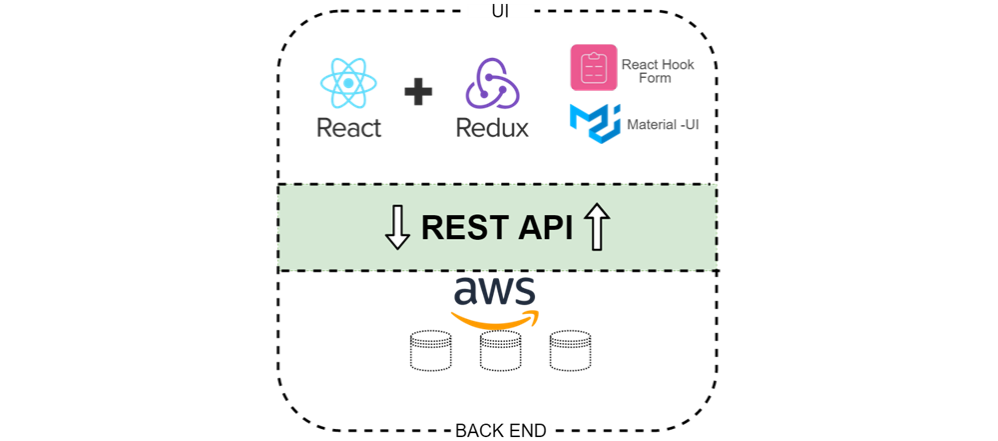
Front end of the SideRep system.

**Figure 3:**
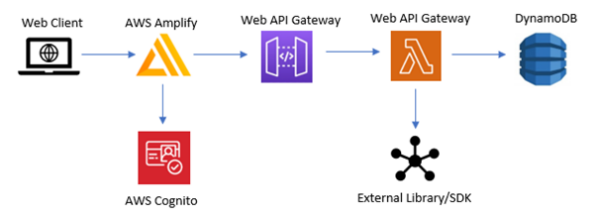
Back end of the SideRep system on Amazon Web Services.

An iterative approach to the development of SideRep was taken, drawing on principles of participatory design [20] and human-centered design [22]. This was in recognition that research of this kind is both socially situated and socially constructed, where end users, as experts of their own experience, should be directly involved in the design decision-making that affects their experience. In the first 2 versions, the systems were developed to run on desktops and various mobile platforms (tested on iPad and Samsung Galaxy 7). The layered architecture of SideRep system allowed some flexibility—the first and second versions were mainly desktop oriented, making use of bespoke JavaScript and designed as a mobile app and desktop application. This dichotomy became a hindrance when moving more heavily to the mobile platform. The development team moved to React library, React Redux toolkit, React Hook Form, Material-UI, Yarn, and Axios for the front end (Figure 2); the third (current) system was hosted and supported by Amazon Web Services (Figure 3).

### User Testing

The first 2 SideRep versions were tested in 2 separate workshops each with 3 end user participants. Participants were recruited from members of the University of Third Age in South Australia, a volunteer organization for people over the age of 50 years. Participants were included if they were taking any regular medications, had access to internet, and agreed to download the app on their phones. The workshops consisted of several stepped activities, where the participants first downloaded the app from the Apple App Store or the Google Play Store, created an account, signed in, and ‘reported’ 3 different and detailed but fictitious adverse events. Each participant was asked to ‘report’ the same adverse effects (ie, headache, skin rash, and indigestion), and therefore, complete the same sequence of events. Participants were observed completing the tasks and were asked a range of semistructured interview questions that aimed to gather data about their experience using the app (eg, ease of use, design, functionality, and language or terminology). A further set of more probing, unstructured, or informal discussion questions were then asked, giving participants the opportunity to share their opinions and ideas for future design development at greater length (eg, the functions they thought might be useful to include or unnecessary features that might otherwise be removed). Observing how participants interacted with the app and talking to them during and after testing helped the researchers understand any perceived difficulties in use or comprehension, so as to incorporate their critical feedback into the third version of the system.

The third (current) version of the SideRep system was tested with consumers who were taking regular medicines. Participants were recruited from people attending public health talks presented by one of the project team members (RL). Participants were invited to trial the SideRep system and complete a modified version of the mHealth app usability questionnaire (MAUQ) [23] after using the system. The MAUQ is an 18-item questionnaire that evaluates the usability of the mobile health app [23]. The MAUQ assessed 3 subscales including ease of use (5 items), interface and satisfaction (7 items), and usefulness (6 items). Each item is scored with a 7-point Likert scale ranging from 0 (strongly disagree) to 7 (strongly agree), with an additional “not applicable” option if the question does not apply to the participants’ experience. Since the third version of the SideRep system was a multiplatform web-based application (ie, not a mobile app), we replaced the word “app” in the MAUQ with “system.”

### Data Analysis

Continuous variables were reported as mean (SD), whereas categorical variables were presented as frequencies (percentages). To aid interpretation of the usability testing data, results were presented both as mean (SD) and as numbers (percentages) of people who answered “strongly agree” (ie, scores of 6 or 7) to each MAUQ item. Data analysis was conducted in Microsoft Excel.

### Ethics Approval

The project was approved by the University of South Australia Human Research Ethics Committee (202532).

## Results

### Content Development

The initial content for the SideRep system was primarily based on findings from our systematic review. Detailed findings of the review has been published elsewhere [21]. Of the 19 questionnaires identified, 15 were for a specific condition or medication, and 4 were general questionnaires applicable to any medication [21]. Two of the generic questionnaires, developed by Jarensiripornkul et al [24] and de Vries et al [25], were considered the most relevant for our study objective. Both questionnaires had a comprehensive list of symptoms categorized in body categories but were lengthy questionnaires. For example, the questionnaire by de Vries [25] took a median of 30 minutes to complete for patients reporting at least one adverse drug event. The length of time needed to complete the questionnaire was considered too long by the project team. The questionnaires were therefore reviewed to extract only questions deemed important and relevant for our study purpose.

After a review by the project team members, the following 5 main questions were extracted for inclusion in the SideRep system: (1) medication(s) recently started, including the date it started (and the date it stopped, if applicable); (2) whether the participants had been taking the medication as prescribed; (3) symptom change experienced in the last 4 weeks, with options classified by body categories; (4) the degree of bothersome symptoms (eg, not at all bothersome, and minimally, moderately, or severely bothersome); and (5) whether the participants had or planned to inform their health care professionals about the symptoms.

### Iterative System Development

**Figure 4:**
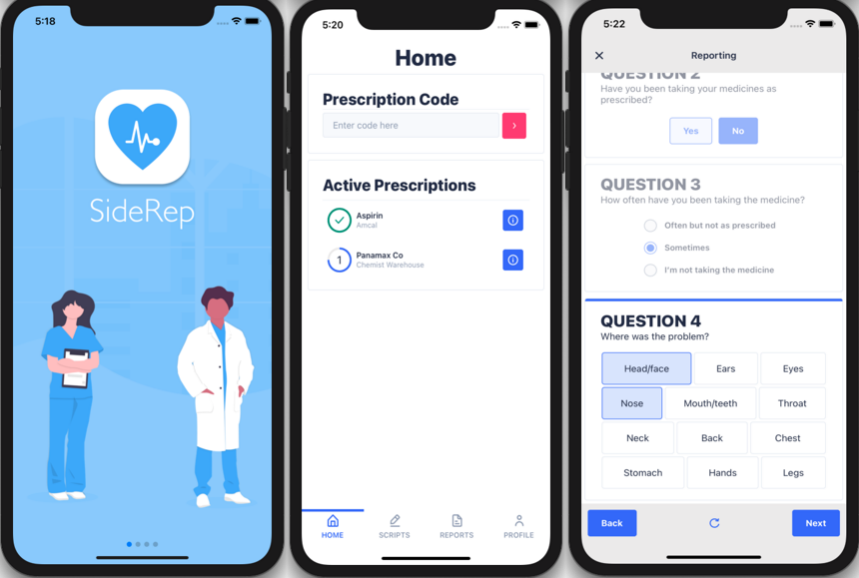
Example screenshots of the second version of SideRep system. Left: main screen; middle: list of medications; right: questions related to reporting of medicine side effects.

The second part of the project involved iterative system development and workshops with small groups of 3 end users each. Participants from the first workshop thought that the SideRep app was easy to use, and they had no problem reporting medicine adverse effects. They provided the investigators with a list of desired features and highlighted design issues that could be improved. This led to the second version, which focused mainly on the functionality and visual design of the app and improving the stability of the app (Figure 4). Feedback was again that the “app was easy to use” and that the longest time taken was for the app download, registration, and log-in. Participants also gave suggestions on the kind of questions to include.

**Figure 5:**
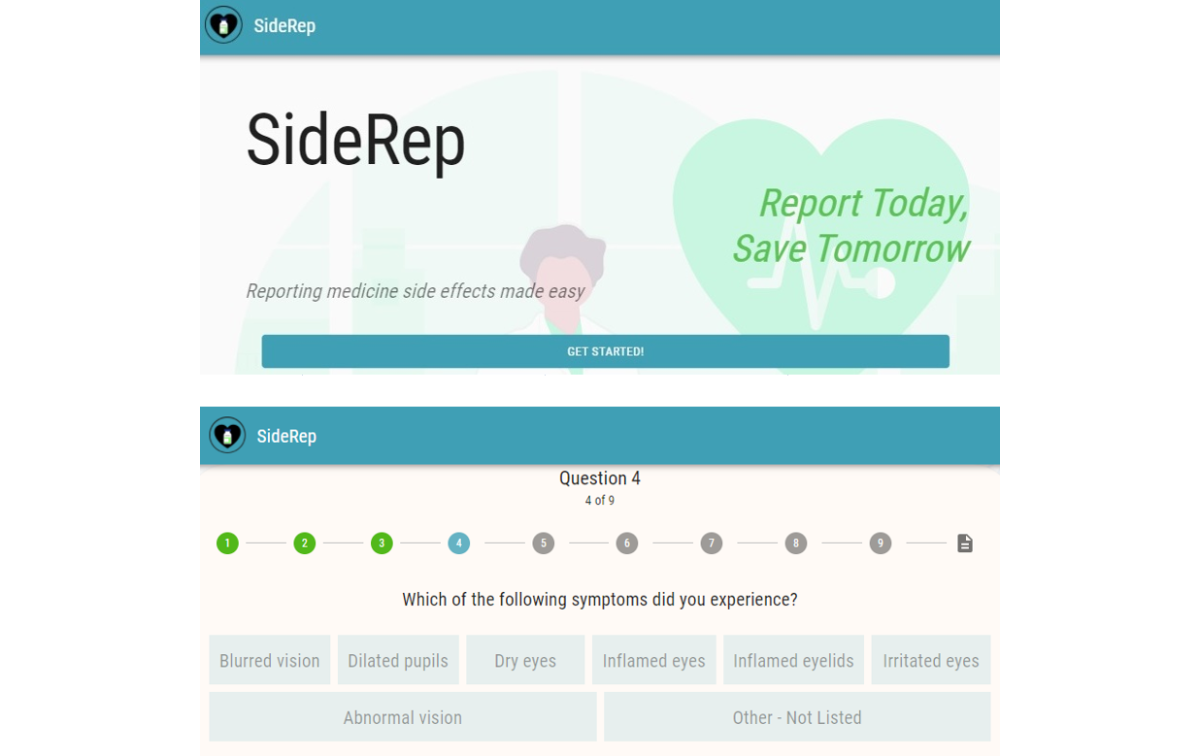
Example screenshots of the third (current) iteration of the SideRep system. Top: main screen; bottom: symptoms listed based on body categories that the participant selected in the previous question.

The third (current) version was developed as a cloud-hosted web-based application with no requirement to download and install an app or to register an account (Figure 5). This immediately mitigated the set of procedures new users found most complex and time-consuming and minimized further risk of error or frustration in user experience. User feedback and consultation within the project team resulted in several minor changes to the final structure and sequence of the questions. There were 8 main questions in the third version of SideRep web system, as follows: (1) what medication(s) was recently used, including the date it started (and the date it stopped, if applicable); (2) symptom change experienced in the last 4 weeks, with options classified by body categories; (3) the degree of discomfort caused by the symptoms; (4) whether the participants had or planned to inform their health care professionals about the symptoms; (5) whether the participants had been taking the medication as prescribed; (6) whether the problem resolved if the participants indicated they stopped taking the medicine; (7) participant characteristics, including age and sex; and (8) free text to include additional information.

### Usability Testing

The third (current) version of the SideRep system was tested with 26 participants. The average age was 45 (SD 11.8) years. A total of 16 (61%) participants were female, 8 (31%) were male, and 2 (8%) preferred not to specify. The SideRep system received an average MAUQ score of 6.1 (SD 1.1), and 79% of all responses were scored 6 or 7, indicating that users generally strongly agree with the usability of the system. When looking at the individual domains, the SideRep system had an average score of 6.3 (SD 0.9) for “ease of use,” with 90% (n=109) of the responses being “strongly agree.” In terms of “interface and satisfaction,” the average score was 6.3 (SD 0.8), and 85% (n=155) of the responses were “strongly agree.” In the “usefulness” domain, the average score was 5.2 (SD 1.4), with 50% (n=43) of the responses being “strongly agree.” The mean (SD) scores and number (%) of “strongly agree” responses for each statement can be seen in Table 1.

**Table 1:** Usability of the SideRep system as assessed using the modified version of the mHealth app usability questionnaire (N=26).

Domain and statements		Mean (SD)	Strongly agree, n/N (%)^a^	Not applicable^b^
**Ease of use**				
	The system was easy to use.	6.5 (0.6)	24/26 (92)	—^c^
	It was easy for me to learn to use the system.	6.5 (0.6)	25/26 (96)	—
	The navigation was consistent when moving between screens.	6.4 (1.1)	23/26 (88)	—
	The interface of the system allowed me to use all the functions (such as entering information, responding to reminders, and viewing information) offered by the system.	6.2 (0.7)	23/26 (88)	—
	Whenever I made a mistake using the system, I could recover easily and quickly.	6.1 (1.2)	14/17 (82)	9
	Overall score	6.3 (0.9)	109/121 (90)	—
**Interface and satisfaction**				
	I like the interface of the system.	6.1 (0.8)	23/26 (88)	—
	The information in the system was well organized, so I could easily find the information I needed.	6.1(0.9)	21/26 (81)	—
	The system adequately acknowledged and provided information to let me know the progress of my action.	6.0 (1.0)	18/26 (69)	—
	I feel comfortable using this system in social settings.	6.6 (0.7)	23/26 (88)	—
	The amount of time involved in using this system has been fitting for me.	6.5 (0.8)	24/26 (92)	—
	I would use this system again.	6.4 (0.7)	22/26 (85)	—
	Overall, I am satisfied with this system.	6.4 (0.6)	24/26 (92)	—
	Overall score	6.3 (0.8)	155/182 (85)	—
**Usefulness**				
	The system would be useful for my health and well-being.	5.2 (1.2)	7/17 (41)	9
	The system improved my access to health care services.	5.1 (1.6)	6/11 (55)	15
	The system helped me manage my health effectively.	5.1 (1.5)	9/17 (53)	9
	This system has all the functions and capabilities I expected it to have.	4.9 (1.3)	9/26 (35)	—
	I could use the system even when the Internet connection was poor or not available.	5.5 (1.6)	5/6 (83)	20
	This system provides an acceptable way to receive health care services, such as accessing educational materials, tracking my own activities, and performing self-assessment.	5.7 (0.8)	7/10 (70)	16
	Overall score	5.2 (1.4)	43/87 (49)	—

^a^N=26 participants except where participants answered “not applicable” to the question.

^b^Number of participants who answered “not applicable” for that statement.

^c^No participants answered “not applicable” to the question.

## Discussion

### Principal Findings

In this paper, we described the development and design of a consumer-friendly digital tool to report medication-related adverse effects and tested the usability of the system in participants who were taking medicines. We drew on principles of participatory design and were guided by a human-centered design methodology, including an iterative design process, and the inclusion of stakeholders, including consumers at key stages of SideRep’s development. Consumer feedback was used to improve the system design and content. The third (current) version of the system was tested in a group of participants. Most users (>85%) strongly agreed that the system was easy to use and were satisfied with the system, with an average score of 6.3 for both “ease of use” (SD 0.9) and “interface and satisfaction” (SD 0.8). About half of the participants strongly agreed that the system was useful, with an average score of 5.2 (SD 1.4) for the “usefulness” domain.

From the earliest applications of user-centered design, the concern has been to learn about and prioritize the needs and preferences of people who will interact with the designed object [22]. This deep understanding is essential to designing digital health systems, like SideRep, that will improve their ease of use and comprehension. One key insight gained through this iterative design process was that an app platform was too limiting and rigid to accommodate the SideRep system. By the nature of smart device apps, users must download software updates to keep the platform active. They also require user log-ins; that can make users reluctant to create a personalized account, and they may also fear for the safety of their data and personal information. These are all factors that may deincentivize people from reporting ADRs. The consumer workshops also revealed that individuals may lack the level of digital literacy needed to complete prerequisite tasks such as finding, downloading, and setting up the app on their smart device, and that this may inadvertently exclude consumers from using the system. In response to these issues and to avoid problematic, confusing, and time-consuming activities that may deter people from reporting ADRs [11,18], SideRep was transitioned from a smart device app to a web-based platform. Importantly, a web-based platform is still accessible on mobile devices as well as laptop and desktop computers; however, websites allow for more fluid, seamless, and responsive updating of the content (eg, adding new medicines, improving clarity of language, and site structure) at a lower cost to developers. This effectively removed the procedural barriers of finding, downloading, and updating an app and enabled an anonymous method of reporting without the burden of individual accounts or log-ins. Furthermore, as web technologies change, a server-based deployment enables the updating and improvement of system performance without the need for end users to be involved. This provides an effective means of future-proofing at a lower overall cost to developers and without the risk of deterring existing or new users. As a result, the usability survey showed high ease of use and participation satisfaction with the reporting system.

Many interventions have been developed to improve the rates of spontaneous ADR reporting to regulatory authorities [13,26,27]. These interventions aim to increase use of spontaneous reporting systems but do not address underlying reasons for low system uptake such as lack of time, difficulty accessing or using the system, negative reporting experience, and lack of feedback on submitted reports [11,18,28]. Many factors may influence acceptability and use of an ADR reporting system, including the number of questions, the way the questions are formulated, the options provided (eg, free text vs menu), and the language used (eg, the use of jargon when the target audience is consumers) [29,30]. In mid-2015, the European Union’s WEB-RADR (or Recognizing Adverse Drug Reactions) project developed and launched a new mobile app to improve reporting of ADRs from patients and health care professionals [31]. The app was developed following focus groups and interviews with potential users. Several desired functionalities were incorporated including 2-way exchange of safety information (ie, users could receive safety alerts and news in addition to reporting ADRs) [31]. Despite this, the uptake was low; between 2015 and 2017, only 838 ADR reports were submitted through the app in countries where the app was launched (ie, United Kingdom, Netherlands, and Croatia) [31]. The effects of making access to the SideRep system more straightforward, that is, without needing to install an app, on system uptake will need to be tested in practice.

### Limitations

We tested the first and second versions of the SideRep system with only a small group of end users (ie, 2 workshops each with 3 end user participants). We had originally intended to conduct face-to-face workshops in the second quarter of 2020, with 10 participants at each workshop. However, due to restrictions following the COVID-19 pandemic, we submitted an ethics amendment to conduct the workshops via web-based videoconferencing. As a result, we had to limit the number of participants at each workshop because it would be too difficult to manage a large number of participants in a web-based workshop. This meant that we received feedback from only a small number of participants for the first and second versions. However, feedback from all consumers during the workshops were generally positive. We did not perform causality assessment to determine whether the adverse effects reported by the consumers were attributed to the medicines, that is, whether or not the effects were ADRs. Causality assessment is an important component for medicine safety signal detection; however, the purpose of our study was to first determine whether an alternative system for reporting medication-related adverse effects was feasible. Thus, causality assessment was considered beyond the scope of our project. Finally, we do not yet know how this system will be implemented in practice and whether it will lead to increased consumer-led ADR reporting to regulatory authorities. It will be important to test how the information reported by consumers can be used by regulatory authorities and health professionals. The next step would include testing the SideRep system in practice using real-world situations.

### Conclusions

The SideRep web-based system for medicine adverse effects reporting is a user-friendly tool developed using an iterative participatory design approach. Future research should include further improving the system, particularly the usefulness of the system, as well as testing the scalability and performance of the system in practice. Successful implementation of the system has the potential to allow for early detection and prevention of medicine-induced harms, and it could increase consumer ADR reporting to regulatory authorities.

## Abbreviations

ADR: adverse drug reaction
MAUQ: mHealth app usability questionnaire.
